# Morphologic and immunophenotypic evidence of in-situ Kaposi's sarcoma

**DOI:** 10.1186/1472-6890-6-7

**Published:** 2006-10-30

**Authors:** Panagiotis A Konstantinopoulos, Bruce J Dezube, Liron Pantanowitz

**Affiliations:** 1 Department of Hematology/Oncology, Beth Israel Deaconess Medical Center, Harvard Medical School, Boston, Massachusetts, USA; 2 Department of Pathology, Baystate Medical Center, Tufts University School of Medicine, Springfield, Massachusetts, USA

## Abstract

**Background:**

The spectrum of Kaposi's sarcoma (KS) has been expanded to include pre-KS lesions.

**Case Presentation:**

We report, for the first time, a case providing direct histological evidence of the development of early (in-situ) KS from mediastinal lymphatic vessels in the setting of chronic lymphedema in an HIV-positive patient. Spindle-shaped and endothelial cells in these early KS-appearing lesions were immunoreactive for HHV8, D2-40 and CD34.

**Conclusion:**

Our findings suggest that HHV8-infected spindle-shaped cells associated with lymphangiogenesis that evolve into KS lesions, acquire from the outset an aberrant mixed vascular and lymphatic endothelial cell phenotype.

## Background

Kaposi's sarcoma (KS) is a vascular neoplasm that may involve mucocutaneous and visceral body sites. KS lesions are comprised of aberrant vessels and spindle-shaped tumor cells that increases in frequency from an early patch and plaque and ultimately establishes a tumor (nodular stage). Several lines of evidence support a lymphatic endothelial origin of Kaposi's sarcoma (KS) [1]. Specifically, KS spindle cells react with monoclonal antibodies to VEGFR-3 (the extracellular domain of the vascular endothelial growth factor-C receptor), which is a marker for lymphatic endothelial cells [2]. The D2-40 antibody is another selective marker of lymphatic endothelium and similarly reacts with KS lesional cells at all stages of progression, supporting the concept that KS originates from a stem cell capable of undergoing lymphatic differentiation [3]. Finally, infection of differentiated blood vascular endothelial cells with human herpesvirus-8 (HHV8) has been demonstrated to induce lymphatic lineage-specific genes with concomitant down regulation of blood vascular genes [4].

The spectrum of KS lesions has been expanded to include pre-KS, a lymphedematous form of KS [5]. We report a case that provides clear histological evidence of the development of such early (in-situ) KS with immunohistochemical verification.

## Case presentation

A 34-year-old homosexual male with acquired immune deficiency syndrome (AIDS)-related KS presented with chylothoraces due to obstruction of his thoracic duct by KS. He had extensive cutaneous lesions on the face, forehead, upper torso, mid-abdomen, left arm and left flank. He had been initially diagnosed with AIDS when he presented with vomiting and bloody diarrhea, and was found via endoscopy to have KS involving the colon. HIV serology was positive and his CD4 T-lymphocyte count at diagnosis was 30 cells/mm3. He was subsequently found via bronchoscopy to have pulmonary KS.

**Figure 1:**
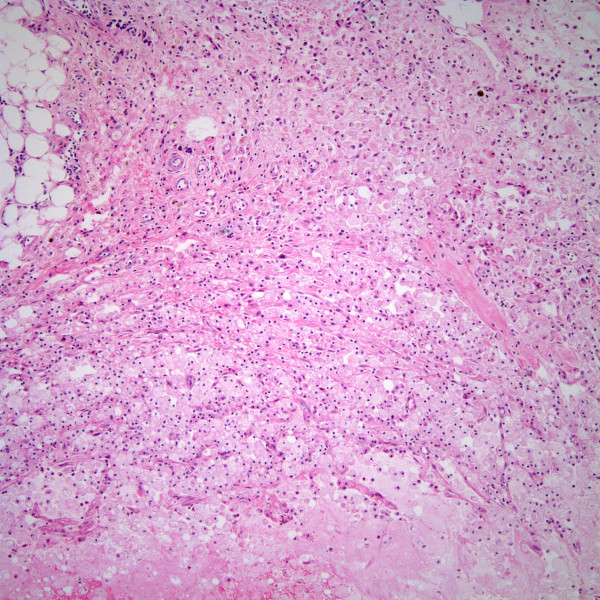
Subcutaneous bile lake with associated xanthogranulomatous reaction (H&E stain, magnification ×100).

Pleuroperitoneal shunt placement and thoracic duct ligation were performed for management of chylothoraces. Since placement of the shunt, he developed ascites with subcutaneous extravassation of lymph that was associated with xanthogranulomatous bile lakes (Figure 1). He received diuretic therapy and medium-chain triglyceride dietary supplementation with only temporary improvement of his ascites. He was requiring paracentesis every 4–6 weeks for resolution of respiratory distress.

He received highly active antiretroviral therapy (stavudine, lamivudine and nelfinavir) and his HIV viral load became undetectable. His KS was treated initially with liposomal daunorubicin and then paclitaxel. He was then switched to SU5416 (an angiogenesis inhibitor) to which he had a temporary response. He was placed on palliative paclitaxel and died of progressive KS about 18 months later.

**Figure 2:**
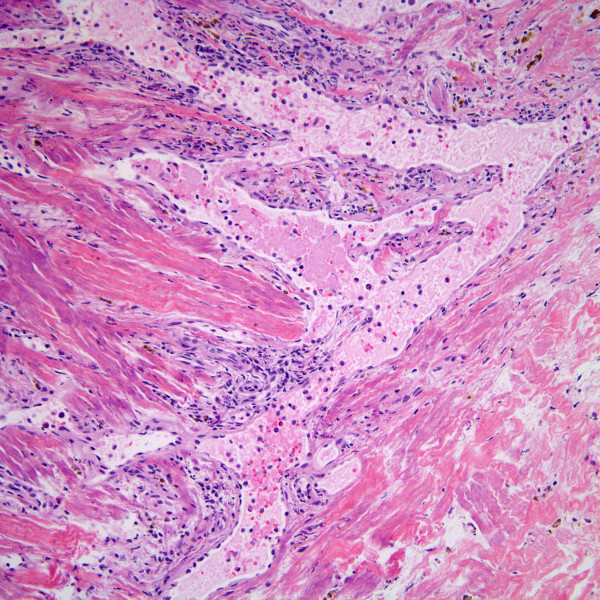
Dilated lymphatics with multifocal areas resembling early KS (H&E stain, magnification ×100).

**Figure 3:**
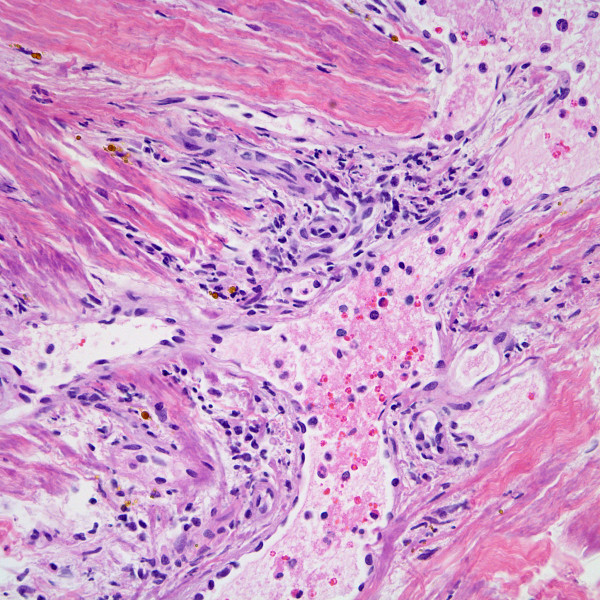
KS in-situ area at higher magnification comprised of small vessels and adjacent spindled cells arising from dilated lymphatics (H&E stain, magnification ×400).

His pleural and lung biopsies showed dilated pleuropulmonary lymphatics (Figure 2) with interstitial pulmonary extravassation of lymph. The biopsy revealed a multifocal increase in spindle-shaped cells with neo-angiogenesis originating from dilated lymphatics, associated with scattered lymphocytes and hemosiderin-laden macrophages, resembling early stage KS (Figure 3).

**Figure 4:**
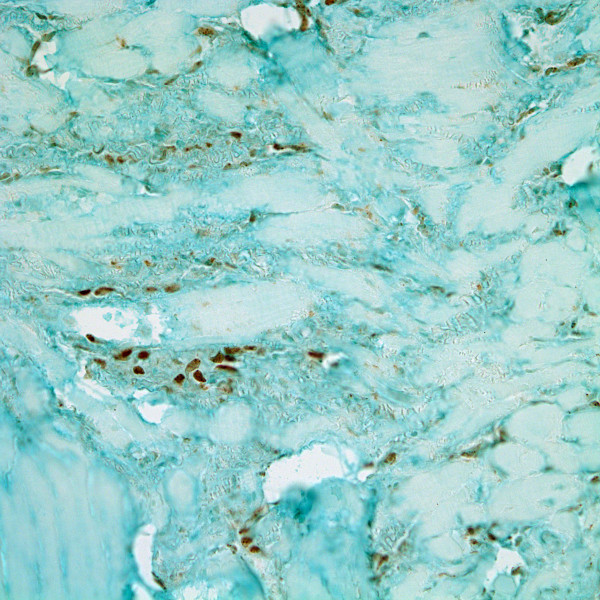
HHV8 positive cells lining dilated lymphatics and focal spindle-shaped cells (LNA-1 immunohistochemical stain; magnification ×400).

**Figure 5:**
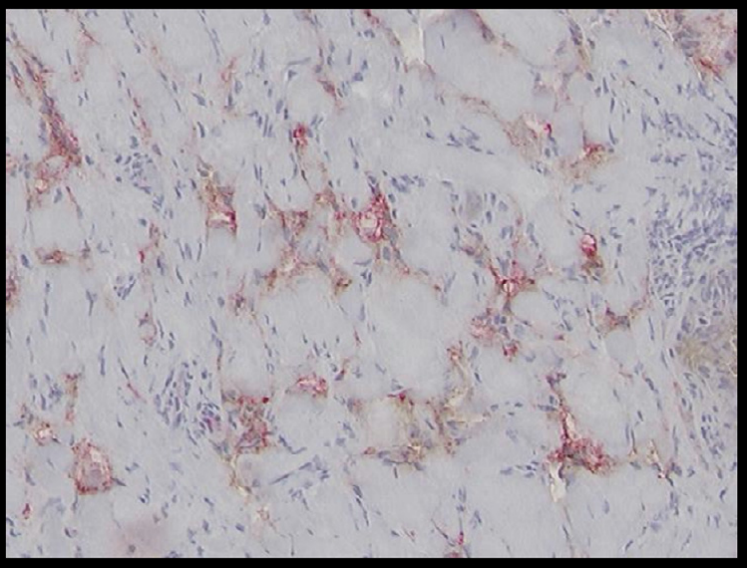
In situ KS lesional cells focally co-express CD34 (brown) and D2-40 (red).

Immunohistochemistry was performed using HHV8 associated Latent Nuclear Antigen-1 (LNA-1; Advanced Biotechnologies, Columbia, MD) monoclonal antibody, as well as dual-color immunostaining with the vascular endothelial marker CD34 (Dako, Carpinteria, CA) and lymphatic specific endothelial marker D2-40 (Signet, Dedham, MA). Spindle-shaped and endothelial cells in these early KS-appearing regions were strongly HHV8 positive (Figure 4) and immunoreactive for both D2-40 and CD34 (Figure 5). Non-lesional lymphatics were HHV8 negative and only D2-40 positive. Native blood vessel endothelium was HHV8 and D2-40 negative, and only CD34 positive.

## Conclusion

We believe that the findings in this case provide direct morphological evidence of the development of an in-situ form of KS directly from lymphatics in the setting of chronic lymphedema. Our results are consistent with previous reports of a cutaneous lymphedematous form of pre-KS [5,6]. In our patient, chronic lymphedema together with HHV8 infection of lymphatic endothelial cells probably led to the development of KS in-situ lesions. This is in concordance with previous reports showing that chronic lymphedema may predispose for local immune incompetence, manifested by the development of KS and Stewart-Treves syndrome (lymphangiosarcoma arising from chronic lymphedema) [7-9]. Chronic lymphedema may occasionally mask the presence of KS while the co-existence of smaller fibroma-like nodules which are frequently associated with chronic lymphedema have the potential to acquire the characteristics of KS [10]. The histological findings in our case were not as exuberant as those reported in the lymphangioma-like variant of KS [11], nor was there any cytological atypia reminiscent of lymphangiosarcoma. Of note, the development of KS from local lymphedema has been reported even in a patient without immunosuppression or HIV infection, who was nevertheless HHV8 seropositive [8]. While bile is well known to have the capability of evoking a xanthogranulomatous reaction, the histopathological findings of subcutaneous xanthogranulomatous bile lakes, as demonstrated in this case, has not been previously reported [12]. Chylothorax is a known but rare manifestation of KS involving the thoracic duct and adjacent mediastinal structures [13,14]. Rare cases of chylous ascites caused by KS have also been noted [15]. Although KS-related chylothorax has been postulated to develop due to metastatic KS of the thoracic duct [16], our findings suggest that chylothorax may arise due to development of in-situ KS in this region. Finally, our findings further indicate that HHV-8-infected spindle-shaped cells that evolve into KS lesions acquire, from the outset, an aberrant mixed vascular and lymphatic endothelial cell phenotype as evident by the coexpression of CD34 and D2-40 on lesional cells.

## Competing interests

The author(s) declare that they have no competing interests.

## Authors' contributions

PAK, BJD and LP were all involved in conception, design, acquisition of data, analysis and interpretation of data and were directly involved in drafting and revising the manuscript. All authors read and approved the final manuscript.

## Pre-publication history

The pre-publication history for this paper can be accessed here:


